# Nanostructured Lipid Carriers Engineered as Topical Delivery of Etodolac: Optimization and Cytotoxicity Studies

**DOI:** 10.3390/ma14030596

**Published:** 2021-01-27

**Authors:** Anna Czajkowska-Kośnik, Emilia Szymańska, Robert Czarnomysy, Julia Jacyna, Michał Markuszewski, Anna Basa, Katarzyna Winnicka

**Affiliations:** 1 Department of Pharmaceutical Technology, Medical University of Białystok, Mickiewicza 2c, 15-222 Białystok, Poland; esz@umb.edu.pl; 2 Departament of Synthesis and Technology of Drugs, Medical University of Białystok, Jana Kilińskiego 1, 15-089 Białystok, Poland; robert.czarnomysy@umb.edu.pl; 3 Department of Biopharmaceutics and Pharmacodynamics, Medical University of Gdańsk, Hallera 107, 80-416 Gdańsk, Poland; julia.jacyna@gumed.edu.pl (J.J.); michal.markuszewski@gumed.edu.pl (M.M.); 4 Department of Physical Chemistry, Faculty of Chemistry, University of Białystok, Ciołkowskiego 1K, 15-245 Białystok, Poland; abasa@uwb.edu.pl

**Keywords:** etodolac, nanostructured lipid carriers (NLC), design of experiment, in vitro release, cytotoxicity

## Abstract

Etodolac (ETD), a nonsteroidal anti-inflammatory drug, exhibits antinflammatory, analgesic, and antipyretic activity. The main type of ETD administration is oral route, which is associated with significant systemic side effects. Nanostructured lipid carriers (NLC), a modern lipid formulation, are non-toxic, biocompatible, can improve the solubility and stability of drugs. Nanostructured lipid carriers (NLC) containing etodolac were prepared by a melt-emulsification and ultrasonication technique. Full factorial design (FFD) was applied to optimize the composition of NLC and their properties such as zeta potential, polidyspersity index, and entrapment efficiency. Formulations consisting of Capryol 90, glicerol monostearate, and Tween 20 displayed particle size below 300 nm, encapsulated drug with efficiency of approximately 87% and prolonged drug release up to 24 h. Stable formulations displayed moderately negative surface charge suggesting their limited ability to interact with skin surface but simultaneously presenting their lower risk to cause cell-membrane disruption. In fact, cytotoxicity assessment using human dermal fibroblasts and human epidermal keratinocytes revealed that etodolac-loaded NLC had no important impact on skin cells viability evaluated in vitro, which might evidence that NLC formulations are safe for dermal delivery. The studies developed were relatively fast and simple, requiring no specialized equipment method to prepare NLC as ETD carriers ensuring better solubility and prolonged drug release.

## 1. Introduction

Etodolac (1,8-diethyl-1,3,4,9-tetrahydropyranol(3,4-b)indole-1-acetic acid, ETD) is a nonsteroidal anti-inflammatory drug belonging to the group of selective cyclooxygenase-2 inhibitors. Apart from anti-inflammatory activity, ETD exerts analgesic and antipyretic activity and is mainly used for osteoarthritis and rheumatoid arthritis. Because of relatively shorter terminal half-life (approximately 7 h), sufficient treatment via oral route requires frequent dosing which may lead to adverse cardiovascular effects, fluid retention, edema, or gastrointestinal problems including bleeding, ulceration, and perforation. Recently, local delivery systems with ETD have gained particular attention with potential to enhance local treatment efficacy and simultaneously reduce systemic adverse effects. ETD is a biopharmaceutical classification system (BCS) class II drug, characterized by poor solubility in water (0.016 mg/mL), but high permeability through a lipophilic barriers (log P 2.5) [1,2,3,4,5,6,7]. To reduce the adverse effect of ETD and to obtain high drug concentration, polymer coatings, carriers that ensure extended release or alternative drug administration route are developed [5,6].

In recent decades, transdermal drug delivery has become a novel strategy as it can improve patient compliance (pain-free and safe application), reduce frequency of administration (elimination of first-pass effect, protection from drug demotion), and provide reduction of side effects [6,7]. It should be stated that one topical preparation (Proxym^®^) is commercially available which is a combined product with several biologically active agents including camphor 4%, linseed oil 3%, menthol 10%, and methyl salicylate 5%. Still there is no topical formulation containing only ETD. A number of research papers have recently drawn attention to the application for topical delivery of ETD, including hydrogel [8], niosomal gel [5], nanosponges [9], liposomes [10], ethosomes [6] and organogel [11]. Nanocrystals, liposomes, nanoemulsions, and lipid nanoparticles have shown promising benefits as modern nanocarriers [12]. Advantages of nanoparticulate carriers include protection of drug (from moisture, enzymes), dose reduction, improved drug bioavailability, and controlled drug release. Lipid drug delivery systems have gained attention because of their characteristics: biocompatibility, simplicity of preparation, economical production, and wide possibilities of application (oral, parenteral, ocular, pulmonary, topical). Nanostructured lipid carriers (NLC) are relatively new variants of lipid vehicles formed by mixing solid and liquid lipids with aqueous surfactant dispersion. NLC are regarded as non-toxic and biocompatible with skin and mucosal tissues, demonstrating occlusion and skin-hydrating effect. After application to the skin, NLC form a mono-layered lipid film on the skin, show an occlusion effect, reduce corneocyte packing, and can improve drug penetration to deeper layers [13,14,15,16,17].

Nanostructured lipid carriers seem to be good carriers for hydrophobic ETD, because they ensure the dissolution of the drug in the lipid phase of the system. Moreover, NLC could reduce the ETD dose by improved bioavailability and controlled drug release. The purpose of the study is to obtain novel NLC made by melt-emulsification and ultrasonication method for topical delivery of ETD. Full factorial design approach was adapted to optimize NLC in terms of entrapment efficiency, particle size, and zeta potential. Additionally, specific efforts have been taken to elaborate the influence of NLC composition on the drug dissolution profile in particular whether these novel carriers increase drug solubility enabling achieving fast therapeutic activity and concurrently help assure prolonged drug release. Cytotoxicity assessments using human dermal fibroblasts and human epidermal keratinocytes were conducted. The studies allowed for the development of new compositions of ETD-loaded lipid carriers and constitute the significant contribution to the future research on the utility of these structures in topical skin preparations. The lipid matrix was created from biocompatible and nontoxic substances, which ensured high ETD solubility. The ETD-NLC formulations were characterized by beneficial properties (low particle size, high zeta potential, and EE%) and provided prolonged drug release.

## 2. Materials and Methods

### 2.1. Materials

Etodolac (ETD) was purchased from Xi’an Health Biochemical Technology Co. (Xi’an, China). Capmul MCM (medium chain mono- and diglycerides) was gifted by Abitec (Jenesville, WI, USA). Capryol 90 (propylene glycol monocaprylate), Labrasol (caprylcaproyl macrogol glycerides), and Compritol 888ATO (glyceryl dibehenate) were obtained from Gattefosse (Nanterre, France). Cremophor EL (polyoxyl-35 castor oil), oleic acid, Span 80 (sorbitan monooleate), Kolliphor RH40 (polyoxyl 40 hydrogenated castor oil), Tween 20 (polyoxyethylene sorbitan monolaurate), Tween 80 (polyoxyethylene sorbitan monooleate), and phosphotungstic acid hydrate were purchased from Sigma Aldrich (Steinheim, Germany). Dynasan 118 (tristearin), Softisan 154 (hydrogenated palm oil), Imwitor 900K (glyceryl stearate) and Witepsol E85 (hard fat) were gifted by IOI Oleo GmBH (Hamburg, Germany). Soybean oil and Miglyol 812 (decanoyl- and octanoyl glycerides) were purchased from Caesar & Loretz (Hilden, Germany). Witepsol H15 (hard fat) was obtained from A.C.E.F. (Fiorenzuola d’Arda, Italy). Rapeseed oil, white wax, and cocoa butter were obtained from Fagron (Kraków, Poland). Almond oil was purchased from Chempol (Wrocław, Poland). Stearic acid, glycerol monostearate, and cetyl alcohol were obtained from Paulika (Trąbki Wielkie, Poland). Linseed oil was purchased from Amara (Kraków, Poland). Potassium dihydrogen phosphate and disodium hydrogen phosphate were provided by Chempur (Piekary Śląskie, Poland). Lecithin, candelilla wax were obtained from Making Cosmetics (Washington, WA, USA). Macadamia nut oil was purchased from Midlands Seed (Ashburton, New Zealand). Sodium acetate anhydrous and acetic acid were purchased from POCH (Gliwice, Poland). Water (prepared by a Milli-Q Reagent Water System (Millipore, Billerica, MA, USA), acetonitrile, and methanol (Merck, Darmstadt, Germany) were of HPLC-grade. All chemicals were of analytical grade. The keratinocytes and fibroblasts cell lines were purchased from American Type Culture Collection (Manassas, VA, USA).

### 2.2. Spectrophotometric and High Performance Liquid Chromatography (HPLC) Analysis

The preliminary test consisted of preparing the ETD spectrum in the 200–300 nm wavelength range. It was observed that ETD showed considerable absorbance at 225 nm and smaller value at 278 nm. As Tween 80 at concentration of 1% in buffer solutions (used in the release test) interfered (shows high absorbance) with wavelength of 225 nm, therefore in the in vitro release test for ETD determinations wavelength λ = 278 nm was applied. Tween 80 showed a negligible absorbance at this wavelength. Quantitative ETD analysis in the release test was carried out by spectrophotometric method using the U-1800 spectrophotomer (Hitachi, Tokyo, Japan). The standard solutions of ETD (5–40 μg/mL) were tested, prepared using phosphate (pH 7.4) and acetate (pH 5.5) buffers, with the addition of 1% Tween 80.

The ETD concentration in solubility tests and ETD entrapment efficiency was investigated by the HPLC method. The HPLC system was Agilent Technologies 1200 Infinity (Agilent, Waldbronn, Germany). The column was Waters Spherisorb ODS2 C-18 (250 × 4.6 mm, 5 μm, Waters, Milford, MA, USA). Analysis was conducted under following conditions: at a flow rate 1.0 mL/min, 20 μL of volume injected sample, at ambient temperature, and at the detection wavelength of 225 nm. The mobile phase contained the mixture of acetonitrile and 0.02 M potassium dihydrogen phosphate buffer pH 6.0 (40:60, *v*/*v*). Standards were analyzed in three replicates. Peak areas were recorded and the calibration graph was obtained. The calibration curve was linear over the range of 1–50 μg/mL (R^2^ = 0.999).

### 2.3. Screening of NLC Components

Several oils were screened for solubility of ETD. Excess of ETD was introduced to vial containing oil/emulsifying agent (2 mL). The obtained mixtures were vortexed and kept at 25 °C ± 0.5 °C in water bath with shaking (200 rpm) for 24 h. The solubility of ETD in emulsifying agents was studied at 40 °C ± 0.5 °C, due to the high viscosity of some surfactants at lower temperature. After reaching the equilibrium the mixtures were centrifuged at 4000 rpm for 15 min. The supernatant after being diluted with acetonitrile was filtered (0.45 μm) and analyzed by HPLC technique as defined in Section 2.2. The ETD solubility of solid lipids was tested by placing 5 mg of drug to 1 g of melted lipid in closed Eppendorf vials. The obtained mixture was shaken in water bath (200 rpm) at 80 °C ± 0.5 °C for 24 h. During this time, the tested sample was visually evaluated for the solubilization of ETD and the next portion of drug was added after dissolution of the previous quantity [18]. The measurements were carried out in triplicate.

The selected liquid and solid lipids were studied for miscibility. The mixtures of solid and liquid lipids (in 1:1 ratio) were stirred at 70 °C ± 0.5 °C using shaking water bath (1 h, 200 rpm). Then, after cooling at ambient temperature the mixtures were analyzed visually in terms of separation of layers in lipid mass [19,20].

### 2.4. Experimental Planning

Based on the solubility study, lipid components that provide the best ETD solubility were selected. Further, the miscibility test showed which combination of solid and liquid lipids gives homogeneous mixture. Subsequently, fractional factorial design, generated using JMP software version 8.0 (SAS, SAS Institute, Cary, NC, USA) was implemented to select factors significantly influencing the preparation of NLC. Miglyol 812 (M) and Capryol 90 (C) were tested as liquid lipids (oil), and stearic acid (SA) and glycerol monostearate (MG) as solid lipids. Concentration of Tween 20, selected as surfactant, and the influence of glycerol addition was also evaluated. Overall concentration of lipids was selected as the fifth factor potentially affecting the studied responses (particle size, PDI, potential zeta and EE%). In the screening studies it was shown that Tween 20 at concentration 1.5 or 2.5% and the addition of 2% glycerol were not found statistically significant (*p* value > 0.05), so for further studies surfactant concentration was constant and set at 2% and glycerol was not added. The ratio of solid to liquid lipid was kept at constant level, namely 70:30.

Then, three independent other variables were chosen for the optimization study: the type of solid and liquid lipid and the concentration of lipid phase. The established dependent variables were particle size, PDI, potential zeta, and EE%. Formulations prepared by the full factorial design (FFD) are presented in Table S1. ETD concentration in analyzed NLC formulations was 0.5%, the ratio of solid to liquid lipid was 70:30, and concentration of Tween 20 was 2%.

### 2.5. Preparation of ETD Nanostructured Lipid Carriers (NLC)

**Figure 1:**
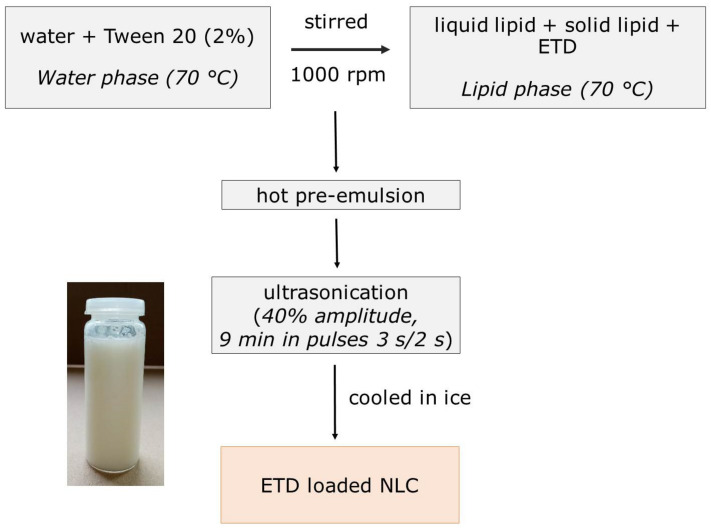
Diagram of nanostructured lipid carriers (NLC) preparation using the melt–emulsification and ultrasonication method and the appearance of etodolac-loaded NLC.

ETD-loaded NLC received by the melt–emulsification and ultrasonication method [21] (Figure 1). In brief, the lipid phase (3% or 5% in total) composed of liquid lipid (M or C) and solid lipid (MG or SA) was melted by heating to 70 °C ± 0.5 °C. ETD (1%) was mixed in the lipid phase until completely dissolved. The aqueous phase containing Tween 20 (2%) was heated to the same temperature as the lipid phase, then added dropwise to the molten lipid phase, and mixed for 10 min at 1000 rpm. The obtained pre-emulsion was ultrasonicated using sonicator (Vibra Cell VCX500, Sonics&Materials, Newtown, CT, USA) at 40% amplitude for 9 min (active every 2 s for 3 s duration) to reduce the particle size. After 5 min of this process, the nanoemulsion was placed in the container with ice, in order to form NLC. The obtained NLC were stored at 4 °C.

### 2.6. Characterization of NLC

Apart from studies described below, all formulations were visually observed to evaluate their consistency and to detect any gelation, creaming, or phase separation process.

#### 2.6.1. Evaluation of Particle Size, PDI, and Zeta Potential

Particle size, polydispersity index (PDI), and zeta potential were examined using Zetasizer Nano ZS 90 (Malvern Instruments, Malvern, UK). Before analysis, water was added to the samples to obtain weak opalescence. The mean particle size and zeta potential were set of three measurements at an angle of 90°.

#### 2.6.2. Determination of Entrapment Efficiency

Entrapment efficiency (EE%) was evaluated by measuring non-encapsulated ETD in the external aqueous phase [22]:

(1) $$\mathrm{EE}\%\;=\;\frac{\mathrm{total}\;\left(\mathrm{mg}\right)\mathrm{drug}\;\mathrm{added}\;\mathrm{to}\;\mathrm{NLC}\;-\;\mathrm{free}\;\left(\mathrm{mg}\right)\mathrm{drug}\;\mathrm{in}\;\mathrm{supernatant}\;\mathrm{after}\;\mathrm{centrifugation}}{\;\mathrm{total}\;\left(\mathrm{mg}\right)\;\mathrm{drug}\;\mathrm{added}\;\mathrm{to}\;\mathrm{NLC}}\;\times \;100\%$$

EE% was measured by centrifugation method using centrifugal filter devices Centrisart 1 (Sartorius, Stonehouse, UK) characterized by nominal molecular weight limit pore size at 10,000. In this method, NLC samples diluted with distilled water was introduced in the upper chamber of a centrifuge tube. Then samples were centrifuged (5000 rpm, 60 min) and after filtration were examined by HPLC method as defined in Section 2.2.

ETD-loaded NLC were dissolved in acetonitrile at 50 °C ± 1 °C in shaking water bath. The samples were filtered (0.45 μm) and ETD was analyzed by HPLC technique and marked as total drug in NLC. The measurement was carried out in triplicate (the average value and SD were calculated).

### 2.7. Preparation the Optimized NLC Formulations

The optimized composition of NLC formulations was determined based on previously described experiment (FFD) (Table 1). In the optimized NLC, the ratio of solid to liquid lipid was 70:30, and the concentration of Tween 20 was 2%. Formulations F9 and F11 were prepared using the melt–emulsification and ultrasonication method (described in Section 2.5). The F10 formulation was prepared by another method—the solvent diffusion technique [23] (F9 and F10 formulations have the same composition, but they were prepared by different method). In this technique the lipids (solid and liquids) and ETD were dissolved in organic solvent (6 mL ethanol) and then quickly dispersed in aqueous solution of 2% Tween 20 under mechanical agitation (1000 rpm) at 70 °C ± 0.5 °C. The stirring was continued until ethanol was evaporated and then the obtained pre-emulsion was ultrasonicated at 40% amplitude for 9 min (active every 2 s for 3 s duration) to reduce the particle size. After 5 min of this process, the nanoemulsion was placed in the container with ice. The obtained NLC were stored at 4 °C ± 0.5 °C.

All NLC formulas were characterized by pH, viscosity, particle size, PDI, potential zeta, and EE%. The in vitro release of ETD was studied and the best kinetic release model for ETD was investigated. The pH of NLC formulations was estimated by pH meter Orion 3 (ThermoScientific, Waltham, MA, USA). The viscosity of formulations was determined by rotational viscometer HAAKE 6plus (ThermoElectron, Karlsruhe, Germany) with using TL5 spindle which rotated at 200 rpm.

### 2.8. Stability of ETD in Different pH

The solutions of ETD (0.3 mg/mL) in acetate (pH 5.5) or phosphate (pH 7.4) buffers with 1% Tween 80 were prepared. The solutions were placed in water bath (100 rpm) at 32 °C ± 0.5 °C. At predefined time points (1, 2, 4, and 24 h), samples were withdrawn and amount of ETD was analyzed by spectrophotometric method, using 278 nm wavelength (as defined in Section 2.2). The aim of this test was to determine the ETD stability in acidic and neutral pH.

### 2.9. Kinetic Study of the In Vitro ETD Release from the Optimized NLC

The in vitro ETD release was tested by the dialysis diffusion technique (the dialysis bags with molecular weight cut off 12,400 Da, Sigma Aldrich, Steinheim, Germany) [24]. Volumes of NLC formulations equivalent to 30 mg of ETD were added into double-folding on both sides dialysis bag and put in 100 mL of release medium. The addition of Tween 80 to the release medium was necessary to provide the sink conditions. Stirring rates were 100 rpm with temperature 32 °C ± 0.5 °C in water bath. The beakers with dialysis bag were covered with parafilm to prevent any evaporation during experimental run. At predefined time points (30 min, 1, 2, 3, 4, 6, and 24 h), samples (1 mL) were withdrawn from the receptor medium and equal amount of the fresh medium was added. The amount of released ETD was analyzed by spectrophotometric method (278 nm). The concentration of ETD was calculated using calibration curve (ETD concentration in the range 5–40 μg/mL). At the same time, the release studies were conducted for ETD suspension (1%) and unloaded NLC. The measurements were performed in three replicates.

The release results were fitted to different mathematical models to explain the release mechanism: zero-order, first-order, Higuchi and Korsmeyer-Peppas model. The best fitted model was chosen based on the highest correlation coefficient (R^2^). The “n” value (Korsmeyer-Peppas model) characterizes the release mechanism (n < 0.45 for Fickian transport and n = 0.45–0.89 for non-Fickian transport) [25].

### 2.10. Morphological Study of the Optimized NLC Formulation

The liquid sample of F9 formulation was applied on a copper grid and then stained by 2% phosphotungstic acid and air dried. The sample morphology was analyzed by transmission electron microscopy (TEM) (TECNAI-G2, 200 kV, HR-TEM, FEI, Eindhoven, The Netherlands).

### 2.11. In Vitro Cytotoxic Assay

The cytotoxicity tests were performed using HDFa (human dermal fibroblasts, adult) and HEKa (human epidermal keratinocytes, adult) culture cell lines were purchased from American Type Culture Collection (Manassas, VA, USA). The viability assay was carried out using tetrazolium salt (MTT), according to Carmicheal et al. [26] with modifications. In the analysis the yellow MTT salt was reduced in viable cells (by active mitochondria) to its purple formazan derivative.

The HDFa and HEKa cell lines were maintained according to the enclosed procedure protocol in Dulbecco’s modified Eagle’s medium (DMEM) and incubated at 37 °C in an atmosphere with 5% CO_2_.

The composition of drug-loaded NLC (ETD-NLC1, ETD-NLC2, ETD-NLC3) and corresponded unloaded formulations (NLC1, NLC2, NLC3) is presented in Table 2. Prior analysis formulations were diluted with using sterile culture medium in a laminar flow cabinet Lamil Plus 13 (Karstulan Metalli Oy, Karstula, Finland) to obtain dilutions in concentration of 50 to 250 mg/mL. Additional control composed of drug diluted in sterile culture medium at concentration of 2.5 mg/mL was prepared. The defined samples volume (25 μL) were introduced to 24-well plates with 1 × 10^5^ per well HDFa or HEKa cells and incubated for 24 or 48 h at 37 °C ± 0.5 °C. The blank was the pure culture medium. The absorbance in living cells was estimated at a wavelength of 570 nm. The results were expressed as the percentage of viable cells as compared to cells incubated only with medium (control).

### 2.12. Statistical Analysis

Quantitative variables were expressed as mean ± standard deviation. Statistical significance was measured using parametric one-way analysis of variance (ANOVA) and Student’s *t*-test or nonparametric methods: the Kruskal–Wallis and Mann–Whitney test using the Statistica 12.5 software (StatSoft, Kraków, Poland). Differences between groups were considered to be significant at *p* < 0.05 and *p* < 0.01. For equality of variance parametric Levene’s test and nonparametric squared rank test were accomplished and the *p*-value > 0.05 indicated that homogeneity of variance was assumed.

## 3. Results

### 3.1. Selection of Components

The ETD solubility is presented in Table S2. Solubility of ETD was found to be higher in medium chain fatty acids (Miglyol 812, Capryol 90, Capmul MCM) as compared to long-chain fatty acids (vegetable oils as almond oil, rapeseed oil, soybean oil, linseed oil, macadamia oil). Among the selected components, the best solubility of ETD in oils was found to be 129.26 mg/g in Capryol 90, 30.78 mg/g in Miglyol 812, and 29.13 mg/g in oleic acid. Capryol 90 and Miglyol 812 which ensured the greatest solubility of ETD and miscibility with solid lipids were selected for further analysis. Tween 20 provided the highest solubility of ETD (30.98 mg/g) and was selected for NLC preparation.

ETD solubility in solid lipids is presented in Figure S1. Among the solid lipids, the best solubility of ETD was noted in cetyl alcohol (20.0 mg/g). ETD solubility in Witepsol H15, glicerol monostearate, and Witepsol E85 was 15 mg/g.

During miscibility experiment it was observed that Miglyol 812 and Capryol 90 with different solid lipids formed homogenous mixture (no separation of layers) upon melting and cooling (Table S3). White wax, cetyl alcohol, Compritol 888ATO, Softisan 154, and Witepsol E85 showed separation into two layers with both liquid oils, whereas stearic acid, glicerol monostearate, Imwitor 900K, Dynasan 118, cocoa butter, and Witepsol H15 showed good homogeneity (no phase separation).

### 3.2. Optimization of NLC Preparation (DOE Approach)

Full factorial design implemented for the optimization studies required preparation of eight different formulations. The obtained results enabled building models for each studied response. They were found to be of a good quality with R^2^ equaled 0.99 each, and p value from 0.005 to 0.043. The lack of fit test proved that built models may be characterized as of a good fit (the lack of fit test was for p value in the range from 0.10 to 0.88).

**Figure 2:**
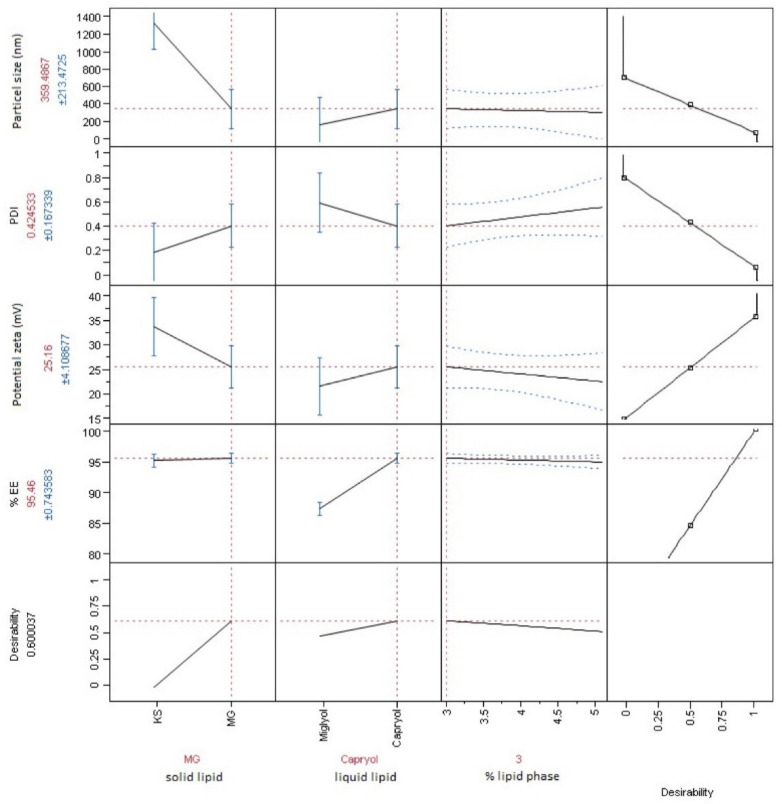
Optimal formulation parameters, using prediction profiler with maximum desirability function.

Figure 2 presents optimal combination of formulation parameters shown in red color. With the use of desirability function, all responses were optimized simultaneously to provide parameters’ settings that will meet criteria for each dependent variable. This solution is favorable in terms of finding a compromise for independent variables, when several responses have to be optimized. Upper limits of acceptance for particle size and PDI were set at 600 nm and 0.7, respectively. Lower acceptable range in terms of EE% was 75% and the absolute value of zeta potential have to be greater than 15 mV. Optimization experiments confirmed preliminary results of a screening study that type of solid lipid is of great importance. Formulations prepared using stearic acid did not meet the requirements (especially in terms of particle size which were above 1 μm). Capryol 90 was also found to be a better component than Miglyol 812, providing higher EE%. On the other hand, the use of Miglyol 812 provided smaller particles. Applied methodology enabled to predict how prepared formulation will be affected by changes in overall lipids concentration, however its influence was found much smaller than the other two factors (type of solid or liquid lipid). The concentration of lipids in NLC equal to 3% leads to provide formulations characterized by slightly higher zeta potential and lower PDI. However, the particle size values and EE% remained at almost the same level. For further studies, the intermediate 4% value (between 3 and 5) of lipids concentration was selected, because of the better ETD solubility in formulations with higher lipids concentration but still maintaining low PDI and high zeta potential.

### 3.3. Assessment of Physicochemical Properties of Optimized ETD-Loaded NLC

Design of experiments (DOE) methodology allowed to develop the optimized ETD-loaded NLC (F9, F10, and F11 formulations). The prepared NLC formulations (F9–F11) were liquid, homogeneous, and milky. All the formulations during storage at 4 °C ± 0.5 °C for 30 days did not show any signs of change in their homogeneity (gelation, phase separation). The F11 formulation showed a slightly yellow color (lecithin content). The pH of the formulations was 4.69, 4.92, and 5.38, consecutively for F9, F10, and F11. The viscosity of NLC dispersion was 2.8 mPas for F9 and 2.4 mPas for F10 formulation. The highest viscosity value equals 3.7 mPas showed a lecithin NLC formulation (F11). After 30 days of storage (4 °C ± 0.5 °C), a small increase in the viscosity of the formulations was noticed: 4.8 mPas in F9, 3.6 mPas in F10, and 8.5 mPas in F11. For studies, the pH value of NLC formulations did not differ significantly during storage for 30 days at temperature 4 °C.

As shown in Table 3, formulation F10 had the highest particle size (627.0 ± 20.8 nm), while the particles size of F9 and F11 were almost similar (273.8 ± 37.6 nm and 245.3 ± 42.2 nm, respectively). Presumably, the addition of ethanol to F10 formulation resulted in the initial dissolution of lipids and dispersion in aqueous phase, then during the evaporation of organic phase the precipitation of lipid phase in the form of larger particles occurred. The PDI values for all tested formulations were similar and ranged from 0.55 to 0.59, which indicates a moderate polydispersity.

The highest value of zeta potential was observed in F11 (−49.1 ± 10.8 mV) because of the presence of soya lecithin in the NLC composition, a lipophilic negative-charge co-surfactant, which may enhance the stability of nanoparticles. The smallest values ranged from −30.7 ± 6.63 mV in formula F10 to −33.2 ± 6.82 mV in formula F9. Zeta potential of all prepared NLC was negative as a consequence of negatively charged glicerol monostearate (MG) and Capryol 90 (C) utilization.

The higher ETD EE% was in formulation F9 (86.5% ± 2.03%), whereas the lower value (79.8% ± 1.43%) was in F10, which was prepared with the addition of ethanol (solvent diffusion method). The smallest EE% value was 67.3% ± 1.98% in F11, with soya lecithin addition.

### 3.4. The Release Test of ETD

**Figure 3:**
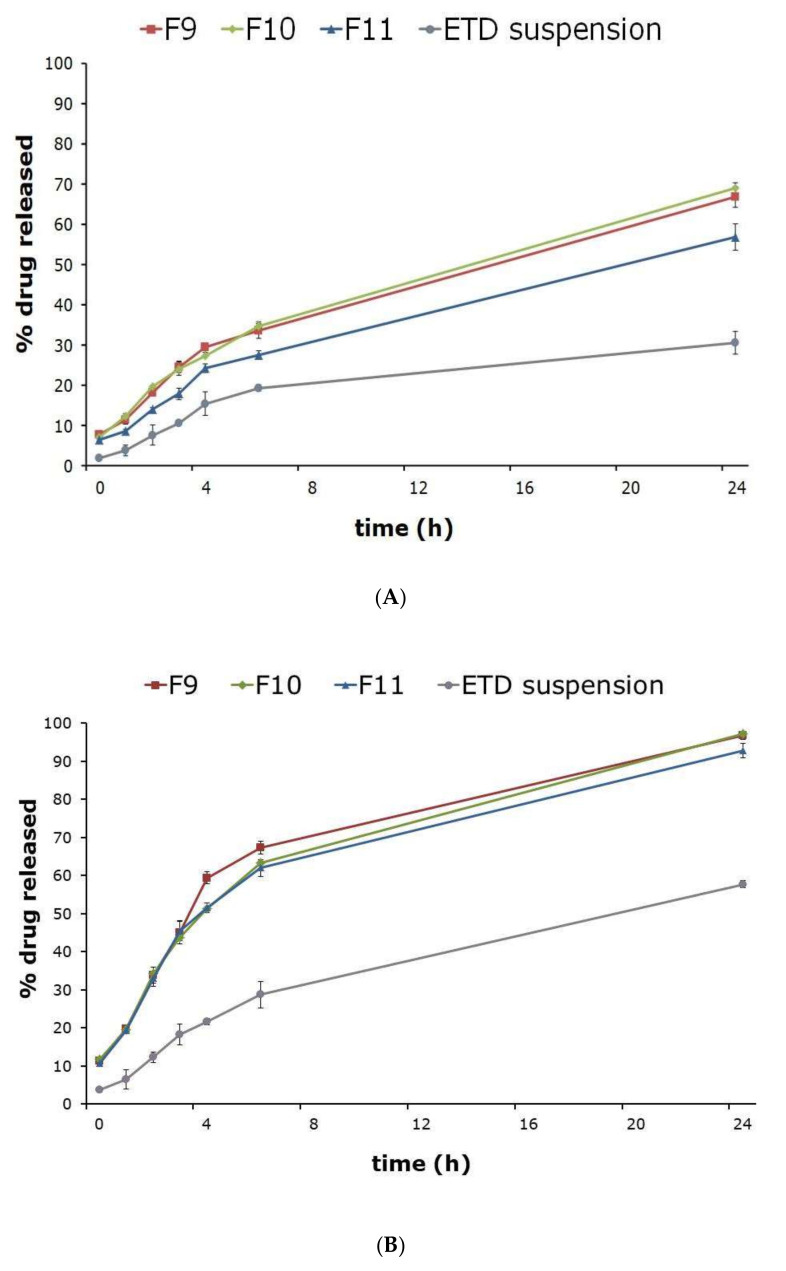
The ETD release from NLC formulations in: (**A**) acetate buffer (pH 5.5), (**B**) phosphate buffer (pH 7.4); containing 1% Tween 80 (mean ± SD; n = 3).

Preliminary tests indicated that ETD is stable for 24 h in the pH 5.5 and 7.4. The release test performed in buffers with different pH (in acetate buffer pH 5.5 and phosphate buffer pH 7.4) showed the effect of the pH value on the amount of ETD released. The cumulative percentage of the released ETD is illustrated in Figure 3. As seen from the graphs, the release of ETD from NLC formulations was faster in phosphate buffer. After 30 min, the release of ETD in acetate buffer was in the range 6.42% to 7.63%, while in the phosphate buffer was in the range 10.78% to 11.67%. After 24 h, ETD release from phosphate buffer was about 100%, whereas in acetate buffer was only in the range 56.89 to 68.91%. The release of 60% of ETD in phosphate buffer was observed after 6 h of the study. On the other hand, the ETD suspension showed 30.50% and 57.73% dissolution in the acetate (pH 5.5) and phosphate (pH 7.4) buffers (with 1% Tween^®^ 80) within 24 h, respectively. The faster release obtained in phosphate buffer was a consequence of its pH value and is associated with better ETD solubility. The effect of pH on ETD solubility in buffers was observed: solubility of ETD was 3.13 mg/mL in phosphate buffer with 1% Tween 80 and only 1.90 mg/mL in acetate buffer with 1% Tween 80. Additionally, ETD release from NLC formulations at both pH values was bi-phasic. The initial burst release (for 4 h) and then long-term (24 h) sustained ETD release from NLC was observed (Figure 3).

The Figure 3A shows that higher percentage of ETD was released from F9 and F10 formulations. Lower amounts of released ETD have been demonstrated for F11. In the case of ETD release in phosphate buffer (Figure 3B), the obtained results were similar for all tested formulations (F9–F11). Interestingly, the results showed that the addition of ethanol as well as soya lecithin to the NLC formulations, has no effect on the ETD release profile.

### 3.5. Kinetic Analysis of ETD Release from Optimized NLC Formulation

**Figure 4:**
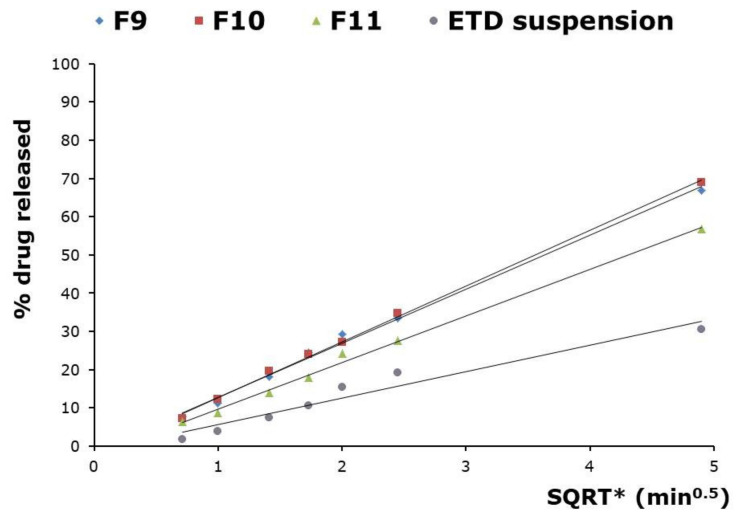
Higuchi diffusion model for the in vitro ETD release from NLC formulations in acetate buffer (pH 5.5) with 1% Tween 80. * SQRT—square root of time.

Various mathematical models were related to ETD release profiles. ETD release from NLC formulations and ETD suspension at pH 5.5 (acetate buffer) showed a fit to Highuchi model with the highest regression coefficient (R^2^) value (0.994 for F9, 0.998 for F10, 0.996 for F11 and 0.994 for ETD suspension) (Table 4). The graphic representation of Highuchi diffusion model is shown in Figure 4. According to this model, the main mechanism of ETD release from NLC formulations was diffusion through the lipid matrix [27]. The lag time for all formulations was between 0.092 and 0.213 h. The ETD release from F9 formulation was the fastest, the slowest release was noted in formula F11.

The release of ETD at phosphate buffer pH 7.4 showed R^2^ value for zero order between 0.747 and 0.928, for first-order between 0.978 and 0.999, for Highuchi model between 0.902 and 0.995, and for Korsmeyer-Peppas model between 0.927 and 0.976. It showed that the best kinetic model for NLC formulations was first order and for ETD suspension was Highuchi model (Table 4). The results of ETD release from all formulations at pH 5.5 and 7.4 showed the n (Korsmeyer-Peppas model) in the range from 0.558 to 0.749, which indicates that release of ETD was a connection of two types: diffusion through the lipid matrix and lipid matrix erosion.

### 3.6. Electron Microscope Examinations

**Figure 5:**
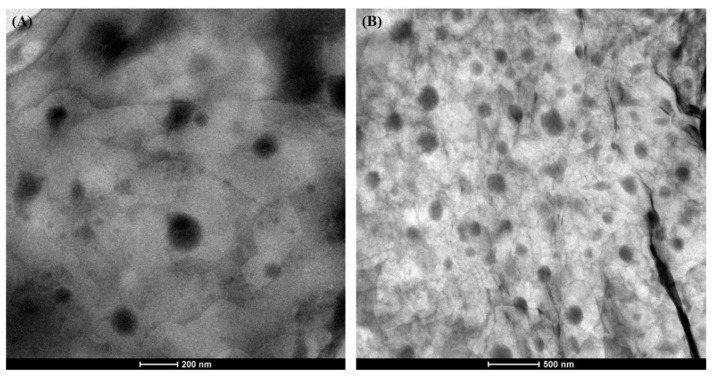
TEM images of F9 formulation. Scale bar represents (**A**) 200 nm and (**B**) 500 nm.

The particles shape of the ETD-NLC (F9 formulation) was estimated by using the electron microscope. The microscopic images showed that particles were spherical, nonadherent, and had a size around 200 nm (Figure 5).

### 3.7. Cytotoxicity Tests

**Figure 6:**
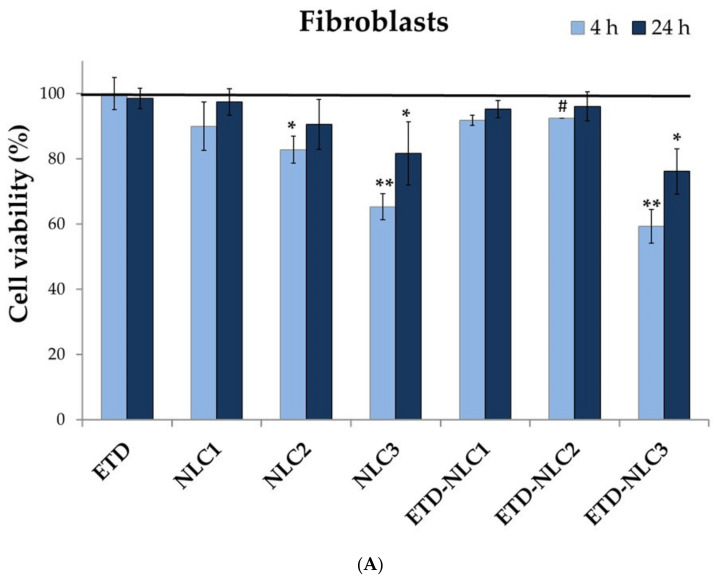

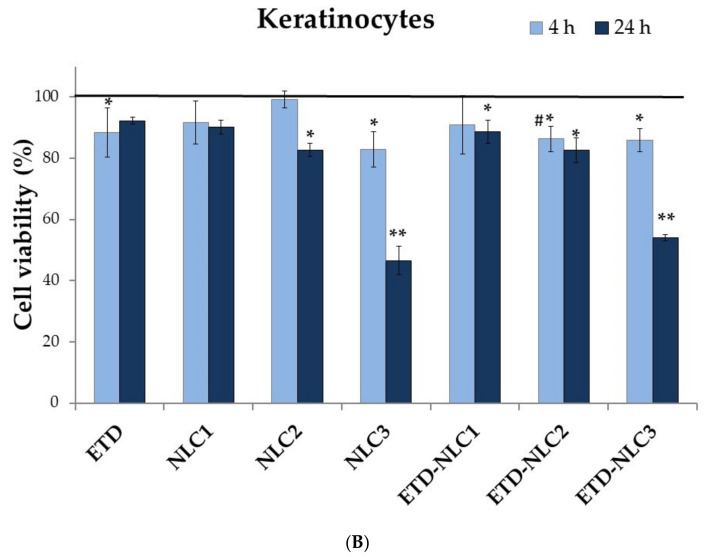
Cell viability of (**A**) fibroblasts and (**B**) keratinocytes, treated with: free ETD, free NLC and ETD-loaded NLC (* significance threshold at *p*-values < 0.05 and ** significance threshold at *p*-values < 0.01 in comparison to control—cells incubated only with medium; # *p* < 0.05 in comparison to NLC without ETD). Data are shown as mean ± SD, n = 3.

The cytotoxicity of ETD, unloaded-NLC (NLC1–NLC3) and ETD-loaded NLC (ETD-NLC1–ETD-NLC2) formulations in two cell lines (HDFa human fibroblasts and HEKa human epidermal kerationocytes) were evaluated (Figure 6). Free ETD can be considered safe, because it did not reduce cell survival below 88% after 24 h. In addition, ETD slightly increased cell viability (from 88.5% to 92.3%) after 24 h incubation in keratinocytes line. The cell viability of fibroblast lines for free ETD was around 100%.

The unloaded nanoparticles at three concentrations of lipid phase (1.25, 2.5, and 6.25 mg/mL) were tested. Figure 6 demonstrated that NLC1 formulation at concentration of 1.25 mg/mL showed no toxic effect on cellular viability (cell viability above 90% was observed after 4 h and 24 h) as compared to control—cells incubated only with medium (*p* values > 0.05). However, an increase in the lipid concentration to 6.25 mg/mL (NLC3 formulation) significantly resulted in lower cell viability. Especially the lower viability (46%) was observed in keratinocytes cell line for NLC3 after 24 h of incubation. ETD-loaded NLC at three concentrations of lipid phase (corresponding to unloaded NLC) and at three concentrations of ETD (0.0125, 0.025, and 0.0625 mg/mL) were studied. Similarly as the unloaded NLC, ETD-NLC formulations with higher lipid concentration caused lower cell viability. As can be seen from the figures, ETD encapsulated in NLC formulations essentially did not affect the viability of the fibroblast and keratinocyte cells (*p* values > 0.05), except for compared to NLC2 and ETD-NLC2 formulations after 4 h of incubation (both cell lines) where slight statistical significance has been observed (*p* = 0.02–0.04). The fibroblasts viability after 24 h of incubation with all unloaded formulations was markedly higher than after 4 h, while the keratinocytes viability after 24 h was lower than after 4 h of incubation (especially for NLC3 formulation in which cell viability after 24 h was 49.6%). In conclusion, the test indicated that pure ETD and NLC formulations in the concentration of lipid phase in the range from 1.25 to 2.5 mg/mL were no cytotoxic (cell viability over 80%), while for the NLC in the lipid concentration 6.25 mg/mL significant decrease in cell viability (even 50%) was observed (*p* values < 0.01).

## 4. Discussion

Nanotechnology has become a widely used field for drug delivery by different route of application. Nanoparticles are characterized as colloidal structures with particle sizes up to 1000 nm. The first generation of lipid nanoparticles are SLN (solid lipid nanoparticles), made of a mixture of solid lipids, surfactants, drug, and water. The main limitation of SLN is the low capacity for the drug, the tendency to polymorphic transformations and the risk of crystallization of active substance during storage. The improved generation of lipid nanocarriers are NLC (nanostructured lipid carriers), which are a mixture of solid and liquid lipids with the addition of surfactants. The presence of liquid lipids allows the nanoparticles to closer higher dose of the active substance and protects it against leakage during storage. NLC are carriers for many medicinal substances, especially they are attractive for drugs that are poorly soluble in water [12,16,28]. The use of NLC is very wide, the literature describes many scientific reports of NLC utility in the drug administration by oral, topical, ocular, parenteral, or pulmonary routes. The use of NLC in gene therapy, chemotherapy, and in the food and cosmetic industries is also described [16,28].

Etodolac (ETD) is one of the non-steroidal anti-inflammatory drugs used as the primary choice in the treatment of rheumatoid arthritis and osteoarthritis. Its main goal is to alleviate the symptoms of the disease, reduce pain due to the inhibition of cyclooxygenase enzymes. ETD is available in oral forms (tablets, capsules) [29,30]. Oral therapy with ETD requires doses of 200–400 mg, every 6–8 h. Limiting the frequency of drug use, its dose, and the possible side effects (gastrointestinal disorders such as peptic ulcer or bleeding) is important issue in ETD therapy [30,31,32]. In order to reduce the side effects associated with the oral route, new ways of drug administration and modern carriers for lipophilic drugs are developed. The goal of effective therapy with ETD is to obtain a constant level of the drug over a longer period of time and to use an efficient low dose [30]. The alternative route of ETD administration is topical or transdermal route. The small size of the nanoparticles allows them to come into close contact with the skin (and to increase drug penetration), moreover some NLC components act as absorption promoters (e.g., oleic acid). The literature shows that NLC can form a film on the skin that prevents water loss and has an occlusive effect (e.g., NLC with a particle size of 200 nm gives a 50% occlusion effect) [16,33]. The study attempted to evaluate NLC as modern vehicles of ETD. These lipid carriers allow the dissolution of the hydrophobic ETD in the lipophilic matrix. The conducted studies included a preliminary analysis of NLC with ETD and allowed to develop research on topical preparations with NLC, assessment of their properties, stability, skin penetration, and safety of use.

Lipids are important components of NLC, which determine the dose of the drug enclosed in the NLC structure, the stability of the system, and the kinetic of drug release. The selection of lipids should be based on the safety of their use (biodegradable, physiologically neutral, non-toxic), and also by their ability of drug dissolution. The evaluation of the drug solubility in lipid solvents is an essential step in pre-formulation studies, determines the drug content in NLC and the encapsulation efficiency [16]. The ratio of solid to liquid lipids is also important, which has crucial impact on the NLC quality (e.g., particle size, zeta potential), stability, and drug release kinetics. NLC are a mixture of liquid and solid lipids and this combination must form a compatible system with no signs of separation. For this purpose, the miscibility of lipid mixtures is assessed (mixture observation for the absence of phase separation) [12,34]. To elaborate the composition of NLC many statistical methods are used [27,31,35,36]. The study used fractional factorial design to plan the experiments and to define selected parameters that determine the obtained NLC. It shows the influence of the most important parameters on the process of NLC preparation, with the optimal number of experiments. The NLC containing various types of lipid and different concentrations of lipid phase were analyzed for determined variables such as particle size, PDI, potential zeta, and EE%. In the study, the preliminary assays such as the solubility test and DOE analysis, allowed to propose the optimal composition of NLC: type of lipids, lipid phase ratio, and final lipid concentration in the NLC structure. It has been shown that the optimal formulations were obtained in the following composition: Capryol 90 (liquid lipid), glycerol monostearate (solid lipid) and Tween 20 (surfactant). The ratio of solid to liquid lipid was 70:30 and the final lipids concentration in NLC was 4%. MCT (medium-chain triglycerides) is widely used as liquid lipid in NLC formulations because of its low molecular weight, high drug solubility, faster digestion, and resistance to oxidation [1,3]. In the study MCT (Capryol 90, Miglyol 812, Capmul MCM) provided better solubility of ETD than LCT (long-chain triglycerides). The solid lipid which ensured high ETD solubility and miscibility with liquid oils was glicerol monostearate. This lipid is non-toxic, non-irritating, widely used in pharmaceutical, cosmetic, and food industries [34]. The surfactant (its type and concentration) affects the NLC quality, being important in obtaining stable systems, in drug solubility and in the release or penetration process [16]. The selection of surfactant in this study was based on ETD solubility tests. The best results was obtained for Tween 20. Tween 20 (polyoxyethylene 20 sorbitan monolaurate) is a water-soluble, non-ionic surfactant with HLB = 16.7. Tween 20 and other polysorbates are considered as non-toxic and non-irritant substances, they are widely used in oral, parenteral, and topical application and in cosmetic or food products [37].

The literature describes many methods of NLC preparation, used both in laboratory conditions and on a larger scale. The frequently described high-pressure homogenization requires the use of specialized equipment and is mainly used in industrial conditions (e.g., in the preparation of nutrition emulsions). All the described methods are based on a similar first step of NLC production. The first stage is the preparation of two phases: the lipid and the water phase. The lipid phase is heated to a temperature above the melting point of the solid lipid and generally in this phase the drug is dissolved. The aqueous phase is formed by water and surfactants and other ingredients (organic solvents, polymers). The next steps of NLC formulation are the effective connection of the water and oil phases [12,16]. In the method applied in this study, the melt-emulsification and ultrasonication method, after the initial mixing (1000 rpm) of both phases and formulating pre-emulsion, the sonication stage (40%) with a cooling action was carried out. This method allowed to obtain NLC dispersions showing no signs of separation and change of consistency.

The physical stability of NLC system is an important parameter evaluated during the long-term storage. The NLC are tested for aggregation, consistency, or separation phase [12]. The composition of NLC (the type of components and their concentrations) and the preparation method have a major influence on the size, zeta potential, and polydispersity index (PDI) of NLC particles. NLC particle size affects the solubility, stability, release, permeation, and biological activity of the active substances. These features of lipid nanocarriers determine the production of stable and good-quality products. Usually particle sizes of NLC range from 10 to 1000 nm. The nanoparticles below 300 nm (which quickly penetrate membranes) are recommended for the treatment of the nervous system and chemotherapy, while particles above 300 nm ensure prolonged drug release. NLC particles size may increase at low surfactant concentration or high drug doses [12,28]. PDI indicates how wide the particle size distribution is, it can range from 0 (perfectly uniform sample) to 1 (highly polydisperse sample). Higher values of this index indicate a wide dispersion in particle size and high polydispersity of the samples [16,38]. Zeta potential, is an expression of the electrokinetic potential in a colloidal distribution, gives an important information about stability of nanoparticles and their tendency to agglomeration [16]. The minimum ±20 mV of zeta potential is required to gain good stability of NLC formulations, however the best value is above ±30 mV [39,40,41]. The drug entrapment efficiency (EE%) is defined by the ratio of the drug associated with NLC to the amount added into the system. It is an important parameter that characterizes NLC in terms of their ability to load the drug. The method of ultrafiltration combined with centrifugation is used to evaluate this parameter. The value of the obtained EE% depends on many factors: the lipophilic properties of drug (lipophilic drugs show high EE% due to their high affinity for the lipid phase), the composition of the lipid phase (it affects for imperfection in lipid matrix), the addition of a surfactant but also the method of preparation [16]. The properties of liquid lipids in NLC increase the imperfections in the crystal structure in lipid matrix which contributes to the EE% increase [12,28,31]. In the study, the optimized NLC formulation (F9) was characterized by 275 nm of particles size, 0.55 of PDI, -33 mV of zeta potential and 86% of EE%. During the NLC preparation it was observed that the addition of lecithin (F11 NLC) had an effect on higher pH and viscosity, lower EE%, particle size, and higher zeta potential. Lecithin is a natural surfactant obtained from egg or soybean. It is not used alone as a surfactant to stabilize NLC (low HLB), but it can be mixed with another surfactants. Other studies have shown that the addition of lecithin may reduce the particle size and PDI of NLC particles [34].

Morphological assessment of formulated NLC was carried out using the TEM (transmission electron microscopy). In the TEM technique, the sample for observation is negative stained (e.g., by using phosphotungstic acid), which provides contrast in the electron microscope. The sample is dried and viewed under an electron microscope (nanoparticles are dark spots on a bright background). Unfortunately, the sample preparation process (drying—dehydration during sample preparation) may lead to changes in the primary morphology of the nanocarriers [16]. The ETD-loaded NLC (F9) developed in the study had the spherical particles with size around 200 nm.

The commonly used method in the assessment of drug release from NLC is the dialysis technique (with using the dialysis bags) [28,30,31]. The release of the drug from NLC depends on many factors, including: drug solubility, type of lipids and surfactants, the drug location in the NLC structure, and the method of preparation. Many reports indicate that drug release from NLC takes place in two stages, characterized by initial burst effect and then sustained release of the active substance. During the preparation of NLC, the drug is separated into both phases (aqueous and oily) at higher temperature. Then, during the cooling, the drug re-enters the lipid phase, that some drug remains on the surface of the nanoparticles (in the outer shell). This fact and liquid lipids which are located at the outer shell of the particles provide the initial drug burst effect in the initial release step. The prolonged drug release takes place from the core of the lipid matrix because of the partitioning between the oil and water phases, diffusion, or matrix erosion. The degradation of the lipid matrix and the kinetics of drug release from NLC depend on the lipid phase properties. Lipids with short fatty chains are degraded faster than LCT [12,16,28]. In the study, the initial burst ETD release and prolonged release (to 24 h) was observed. After 24 h, the ETD release was about 100% in phosphate buffer and about 60% in acetate buffer. The kinetic mechanism of ETD release from NLC formulation was Highuchi model in acetate buffer and first order model in phosphate buffer. The “n” values (Korsmeyer-Peppas) indicated the combination of two release mechanism: drug diffusion and degradation of lipid matrix. The prolonged drug release from NLC formulations and similar results in ETD release were obtained in other studies [10,11,30,31,42,43,44,45].

NLC as carriers allows to increase the solubility of the hydrophobic drugs and they can affect the stability and the drug release. The main component of NLC—lipid phase must be selected for their safety. The cytotoxicity tests allow for the initial assessment of NLC systems. In the study two lines of cells were used: fibroblasts and keratinocytes, and the cytotoxicity evaluation was made by the MTT test. In general, NLC formulations can be considered as non-toxic carriers for drugs [36,46]. However, the other experiments showed that the low particles size (<300 nm) and the lipophilic nature of NLC formulation can affect their penetration into cells and induce stronger effect on the cancer cells viability. These features can be used in cancer therapy [42,47,48]. Performed studies indicated that viability of cells is associated with the lipid concentrations. NLC formulations in the concentration of lipid phase up to 2.5 mg/mL were no cytotoxic in HDFa (human dermal fibroblasts, adult) and HEKa (human epidermal keratinocytes, adult) culture cell lines, while in the concentration 6.25 mg/mL significant decrease in the keratinocytes viability even 50% was observed (*p* values < 0.01).

NLC properties (particle size, zeta potential, EE%, drug release, toxicity) significantly depend on lipid components (type of lipids, solid to liquid lipid ratio, concentration of the lipid phase in NLC). Despite the fact that there are some reports on the use of lipid carriers for ETD, in this study NLC for ETD with optimal properties as particle size, zeta potential, EE%, ensuring bi-phasic release (burst effect and prolonged ETD release) were designed. The new composition (mixture of Capryol 90 as liquid lipid and glycerol monostearate as solid lipid) and the simple method of preparation (the melt–emulsification and ultrasonication method) make formulated carriers promising ETD delivery systems which can be further utilized in pharmaceutical formulations, like topical semi solid dosage forms.

## 5. Conclusions

In conclusion, NLCs containing ETD were received by the melt-emulsification and ultrasonication method. DOE methodology proved to be an efficient strategy to find the optimal factors settings for multi-response process, which is optimization of preparation procedure of NLC. The use of fractional factorial design was favorable in terms of minimizing the overall number of experiments needed to be performed. The optimized NLC formulation (F9) consists of Capryol 90 (liquid lipid), glycerol monostearate (solid lipid), and Tween 20 (surfactant). ETD release from NLC formulations achieved prolonged dissolution profile. The results of cytotoxicity study suggest that NLC formulations at lipid concentrations below 6.25 mg/mL seem to be safe for dermal use. The presented results imply a possible application of designed ETD-loaded NLC in pharmaceutical formulations. However, further assays are necessary to evaluate the utility of these carriers incorporated in different final drug forms, e.g., in semi-solid forms for topical use. It can be concluded that the designed nanostructured lipid carriers are characterized by many beneficial parameters (pH, EE%, particle size, PDI, zeta potential, drug release) comparable with the properties of other modern candidates for etodolac (nanosponges, ethosomes, or niosomal formulations). Further studies on the topical formulations with ETD-loaded NLCs are planned and will be continued, especially in terms of semi-solid forms preparations and their rheological and adhesive properties, stability assessment, and skin permeation evaluation.

## Figures and Tables

**Table 1:** Composition of optimized etodolac (ETD)-NLC formulations.

Formulation	ETD (%)	Solid Lipid	Oil	Lipids (%)	Additional Component	Water (g)
F9	1.0	MG	C	4.0	-	up to 30.0
F10	1.0	MG	C	4.0	Ethanol (6 mL)	up to 30.0
F11	1.0	MG	C	4.0	Soya Lecithin (3%)	up to 30.0

Abbreviations: MG, glycerol monostearate; C, Capryol 90.

**Table 2:** Composition of formulations to cytotoxicity assay.

Formulation	Composition (%)					NLC Concentration after Dilution with Culture Medium (mg/mL)	Final NLC/ETD Concentration in Plate (mg/mL)
	ETD	C	MG	Tween 20	Water Phase		
ETD	1.0	-	-	-	99.0	0	-/0.0625
NLC1	-	1.2	2.8	2.0	94.0	50	1.25/-
NLC2						100	2.5/-
NLC3						250	6.25/-
ETD-NLC1	1.0	1.2	2.8	2.0	93.0	50	1.25/0.0125
ETD-NLC2						100	2.5/0.025
ETD-NLC3						250	6.25/0.0625

Abbreviations: C, Capryol 90; MG, glycerol monostearate.

**Table 3:** Characteristics of ETD-NLC formulations (mean ± SD; n = 3).

Formulation	Visually Observation	pH	Viscosity * (mPas)	Total Drug in NLC (%)	EE%	Particle Size (nm)	PDI	Zeta Potential (mV)
F9	liquid, milky, homogenous	4.69 ± 0.05	2.80 ± 0.15	108.97 ± 3.81	86.50 ± 2.03	273. ± 37.6	0.557 ± 0.04	−33.2 ± 6.8
F10	liquid, milky, homogenous	4.92 ± 0.11	2. ± 0.09	103.63 ± 4.11	79.80 ± 1.43	627.0 ± 20.8	0.580 ± 0.02	−30.7 ± 6.6
F11	liquid, slightly yellow, homogenous	5.38 ± 0.08	3.70 ± 0.11	98. ± 4.66	67.30 ± 1.98	245.3 ± 42.2	0.595 ± 0.06	−49.1 ± 10.8

* viscosity was analyzed by Viscotester 6 Plus (Thermo Scientific, Karlsruhe, Germany) using a rotor TL5 (25 °C ± 1 °C).

**Table 4:** Drug release kinetics for ETD-loaded NLC formulations in acetate (pH 5.5) and phosphate (pH 7.4) buffers, with 1% Tween 80.

Formulation	Kinetic Model					
	Zero Order	First Order	Highuchi		Korsmeyer-Peppas	
	R^2^	R^2^	R^2^	LT (h)	R^2^	n
**Acetate Buffer (pH 5.5)**						
F9	0.927	0.987	0.994	0.092	0.989	0.570
F10	0.939	0.987	0.998	0.128	0.989	0.578
F11	0.942	0.983	0.996	0.213	0.991	0.584
ETD Suspension	0.819	0.883	0.994	0.196	0.934	0.749
**Phosphate Buffer (pH 7.4)**						
F9	0.747	0.991	0.902	0.345	0.927	0.571
F10	0.815	0.999	0.947	0.259	0.951	0.558
F11	0.782	0.984	0.927	0.333	0.934	0.563
ETD Suspension	0.928	0.978	0.995	0.398	0.976	0.727

Abbreviations: R^2^, regression coefficient; LT, lag time; n, value characterize release mechanism.

## Data Availability

The data supporting reported results are available on request from the corresponding author.

## Supplementary Materials

The following are available online at https://www.mdpi.com/1996-1944/14/3/596/s1, Figure S1: ETD solubility in solid lipids (mean ± SD; n = 3), Table S1: Composition of the analyzed NLC formulations, Table S2: Solubility of ETD in oils and surfactants (mean ± SD; n = 3), Table S3: Miscibility of solid and liquid lipids.

## References

[1] O’Neil M. (2006). The Merc Index.

[2] Council of Europe (2007). European Pharmacopoeia.

[3] Drug Bank. https://www.drugbank.ca.

[4] Trissel L.A. (2012). Trissel’s Stability of Compounded Formulations.

[5] Shilakari A.G., Asthana A., Singh D., Sharma P.K. (2016). Etodolac containing topical niosomal gel: Formulation development and evaluation. J. Drug Deliv..

[6] Madhavi N., Sudhakar B., Suresh Reddy K.V.N., Vijaya Ratna J. (2018). Pharmacokinetic and pharmacodynamic studies of etodolac loaded vesicular gels on rats by transdermal delivery. DARU J. Pharm. Sci..

[7] Lee H., Song C., Baik S., Kim D., Hyeon T., Kim D.H. (2017). Device-assisted transdermal drug delivery. Adv. Drug Deliv. Rev..

[8] Tas C., Ozkan Y., Okyar A., Savaser A. (2007). In vitro and ex vivo permeation studies of etodolac from hydrophilic gels and effect of terpens as enhancers. Drug Deliv..

[9] Abbas M.M., Rajab N.A. (2019). Preparation and characterization of etodolac as a topical nanosponges hydrogel. Iraqi J. Pharm. Sci..

[10] Chintala P.K., Padmapreetha J. (2014). Formulation and in vitro evaluation of gel containing ethosomes. Int. J. Pharm. Sci. Res..

[11] Pawar S., Jahagirdar A., Kolkar D., Patil M., Udavant P., Kshirsagar S. (2015). Investigation of potential of organogel carrying etodolac for anti-inflammatory activity. Pharm. Biol. Eval..

[12] Khosa A., Reddi S., Saha R.N. (2018). Nanostructured lipid carriers for site-specific drug delivery. Biomed. Pharmacother..

[13] Czajkowska-Kośnik A., Szekalska M., Winnicka K. (2019). Nanostructured lipid carriers: A potential use for skin drug delivery systems. Pharm. Rep..

[14] Beloqui A.B., Solinis M.A., Rodriguez-Gascón A., Almeida A.J., Preat V. (2016). Nanostructured lipid carriers: Promising drug delivery systems for future clinics. Nanomed. Nanotechnol..

[15] Purohit D.K., Nandgude T.D., Poddar S.S. (2016). Nano-lipid carriers for topical application: Current scenario. Asian J. Pharm..

[16] Chauhan I., Yasir M., Verma M., Singh A.P. (2020). Nanostructured lipid carriers: A groundbreaking approach for transdermal drug delivery. Adv. Pharm. Bull..

[17] Jaiswa P., Gidwani B., Vyas A. (2016). Nanostructured lipid carriers and their current application in targeted drug delivery. Artif. Cells Nanomed. Biotechnol..

[18] Joshi M., Patravale V. (2006). Formulation and evaluation of nanostructured lipid carrier (NLC)—Based gel of valdecoxib. Drug Dev. Ind. Pharm..

[19] Iqbal B., Ali J., Baboota S. (2018). Silymarin loaded nanostructured lipid carrier: From design and dermatokinetic study to mechanistic analysis of epidermal drug deposition enhancement. J. Mol. Liquids.

[20] Negi L.M., Jaggi M., Talegaonkar S. (2014). Development of protocol for screening the formulation components and the assessmnet of common quality problems of nanostructured lipid carriers. Int. J. Pharm..

[21] Almeida H., Lobão P., Frigeriob C., Fonseca J., Silva R., Quaresma P., Sousa L.J.M., Amaral M.H. (2016). Development of mucoadhesive and thermosensitive eyedrops to improve the ophthalmic bioavailability of ibuprofen. J. Drug Deliv. Sci. Technol..

[22] Sütö B., Berkó S., Kozma G., Kukovecz Á., Budai-Szücs M., Erös G., Kemény L., Sztojkov-Ivanov A., Gaspar R., Csányi E. (2016). Development and ibuprofen-loaded nanostructured lipid carrier-based gels: Characterization and investigation of in vitro and in vivo penetration through the skin. Int. J. Nanomed..

[23] Jagdevappa P., Prashant G., Ravindra K., Sachin J., Satish M., Meghanath S. (2013). Application of solid lipid nanoparticle in novel drug delivery system. Br. Biomed. Bull..

[24] Yuan H., Wang L.L., Yiu J., Du Y.Z., Hu F.Q., Zeng S. (2007). Preparation and characteristics of nanostructured lipid carriers for control-releasing progesterone by melt-emulsification. Colloids Surf. B.

[25] Costa P., Lobo J.M.S. (2001). Modelling and comparison of dissolution profiles. Eur. J. Pharm. Sci..

[26] Carmichael J., DeGraff W.G., Gazdar A.F., Minna J.D., Mitchell J.B. (1987). Evaluation of a tetrazolium-based semiautomated colorimetric assay: Assessment of chemosensitivity testing. Cancer Res..

[27] Swidan S.A., Mansour Z.N., Mourad Z.A., Elhesaisy N.A., Mohamed N.A., Bekheet M.S., Badawy M.A., Elsemeiri M.M., Elrefaey A.E., Hassaneen A.M. (2018). DOE, formulation, and optimization of repaglinide nanostructured lipid carriers. JAPS.

[28] Haider M., Abdin S.M., Kamal L., Orive G. (2020). Nanostructured lipid carriers for delivery of chemotherapeutics: A review. Pharmaceutics.

[29] Kesharwani D., Paliwal R., Satapathy T., Paul S.D. (2019). Rheumatiod arthritis: An updated overview of latest therapy and drug delivery. J. Pharmacopunct..

[30] Salah S., Mahmoud A.A., Kamel A.O. (2017). Etodolac transdermal cubosomes for the treatment of rheumatoid arthritis: Ex vivo permeation and in vivo pharmacokinetic studies. Drug Deliv..

[31] Gill V., Nanda A. (2020). Preparation and characterization of etodolac bearing emulsomes. Int. J. App. Pharm..

[32] Kumar S.S., Masilamani K., Srinivas S.S., Ravichandiran V. Formulation and physico-chemical evaluation of ethylcellulose microspheres containing etodolac. Proceedings of the International Conference on Biotechnology and Pharmaceutical Sciences.

[33] Fernandes A.V., Pydi C.R., Verma R., Jose J., Kumar L. (2020). Design, preparation and in vitro characterizations of fluconazole loaded nanostructured lipid carriers. Braz. J. Pharm. Sci..

[34] Azar F.A.N., Pezeshki A., Ghanbarzadeh B., Hamishehkar H., Mohammadi M. (2020). Nanostructured lipid carriers: Promising delivery systems for encapsulation of food ingredients. J. Agric. Food Res..

[35] Siahdasht F.N., Farhadian N., Karimi M., Hafizi L. (2020). Enhanced delivery of melatonin loaded nanostructured lipid carriers during in vitro fertilization: NLC formulation, optimization and IVF efficacy. RSC Adv..

[36] Yu S., Tan G., Liu D., Yang X., Pan W. (2017). Nanostructured lipid carrier (NLC)-based novel hydrogels as potential carriers for nepafenac applied after cataract surgery for the treatment of inflammation: Design, characterization and in vitro cellular inhibition and uptake studies. RSC Adv..

[37] Rowe R.C. (2009). Handbook of Pharmaceutical Excipients.

[38] Danaei M., Dehghankhold M., Ataei S., Hasanzadeh D.F., Javanmard R., Dokhani A., Khorasani S., Mozafari M.R. (2018). Impact of particle size and polydispersity index on the clinical applications of lipidic nanocarrier systems. Pharmaceutics.

[39] Xu R. (2008). Progress in nanoparticles characterization: Sizing and zeta potential measurement. Particuology.

[40] Tamjidi F., Shahedi M., Varshosaz J. (2013). Nanostructured lipid carriers (NLC): A potentail delivery system for bioactive food molecules. Innov. Food Sci. Emerg. Tech..

[41] Üner M. (2006). Preparation, characterization and physico-chemical properties of solid colloidal drug carrier systems. Pharmazie.

[42] Swidan S.A., Ghonaim H.M., Samy A.M., Ghorab M.M. (2016). Efficacy and in vitro cytotoxicity of nanostructure lipid carriers for paclitaxel delivery. JAPS.

[43] Jia L., Shen J., Zhang D., Duan C., Liu G., Zheng D., Tian X., Liu Y., Zhang Q. (2012). In vitro and in vivo evaluation of oridonin-loaded long circulating nanostructured lipid carriers. Int. J. Biol. Macromol..

[44] Liu J.L., Zhang W.J., Li X.D., Yang N., Pan W.S., Kong J., Zhang J.S. (2016). Sustained-release genistein from lipid carrier suppresses human lens epithelial cell growth. Int. J. Ophthalmol..

[45] Almousallam M., Moia C., Zhu H. (2015). Development of nanostructured lipid carrier for dacarbazine delivery. Int. Nano Lett..

[46] Barbosa R.M., Silva C.M.G., Bella T.S., Araúji D.R., Marcato P.D., Durán N., Paula E. (2013). Cytotoxicity of solid lipid nanoparticles and nanostructured lipid carriers containing the local anesthetic dibucaine designed for topical application. J. Phys. Conf. Ser..

[47] Bang K.H., Na Y.K., Huh H.W., Hwang S.J., Kim M.S., Kim M., Lee H.K., Cho C.W. (2019). The delivery strategy of paclitaxel nanostructured lipid carrier coated with platelet membrane. Cancers.

[48] Sabzichi M., Mohammadian J., Khosroushahi A.Y., Bazzaz R., Hamishehkar H. (2016). Folate-targeted nanostructured lipid carriers (NLCs) enhance (letrozol) efficacy in MCF-7 breast cancer cells. Asian Pac. J. Cancer Prev..

