# Supplementary material for: Nanostructured Lipid Carriers Engineered as Topical Delivery of Etodolac: Optimization and Cytotoxicity Studies
Source: Materials (Basel). 2021 Jan 27;14(3):596. doi: 10.3390/ma14030596 (PMC7866147; doi:10.3390/ma14030596)
Supplement: Supplementary file 1 [file materials-14-00596-s001.pdf]

materials

Spplementary Materials: Nanostructured Lipid Carriers as Modern Approach for Etodolac Delivery

Anna Czajkowska-Kośnik, Emilia Szymańska, Robert Czarnomysy, Julia Jacyna, Michał Markuszewski, Anna Basa and Katarzyna Winnicka

Table 1. Composition of the analyzed NLC formulations.

Formulation	Solid Lipid	Oil	Lipids (%)
F1	MG	M	3.0
F2	MG	M	5.0
F3	SA	C	5.0
F4	SA	M	3.0
F5	MG	C	5.0
F6	SA	M	5.0
F7	MG	C	3.0
F8	SA	C	3.0

Abbreviations: MG, glycerol monostearate; SA, stearic acid; M, Miglyol 812; C, Capryol 90.

Table 2. Solubility of ETD in oils and surfactants (mean ± SD; n=3).

Solvent	Solubility (mg/g)
**Oil**	
Miglyol 812	**30.78 ± 1.05**
Capryol 90	**129.26 ± 3.67**
Capmul MCM	14.85 ± 1.57
Oleic acid	29.13 ± 1.29
Almond oil	12.27 ± 0.30
Rapeseed oil	9.86 ± 1.31
Soybean oil	12.98 ± 1.02
Linseed oil	11.26 ± 0.33
Macadamia oil	12.61 ± 0.55
**Surfactant**	
Cremophor EL	11.88 ± 0.19
Kolliphor RH40	12.63 ± 0.70
Labrasol	25.67 ± 0.21
Tween 20	**30.98 ± 1.19**
Tween 80	28.41 ± 0.21
Span 80	15.89 ± 0.12
Soya lecithin	0.38 ± 0.02

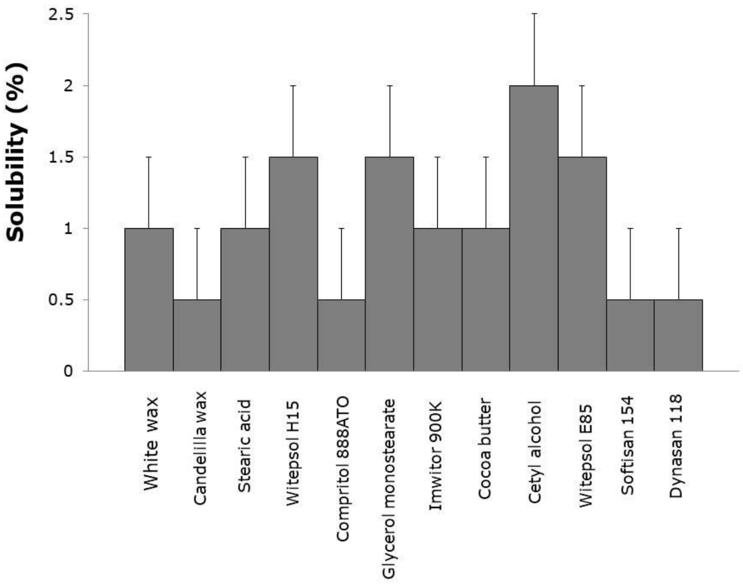

Figure 1. ETD solubility in solid lipids (mean ± SD; n=3).

Table 3. Miscibility of solid and liquid lipids.

**Solid lipid**	**Liquid lipid**	
	**Miglyol 812**	**Capryol 90**
White wax	mild phase separation	mild phase separation
Candelilla wax	homogenous	phase separation
Stearic acid	homogenous	homogenous
Cetyl alcohol	mild phase separation	phase separation
Glycerol monostearate	homogenous	homogenous
Compritol 888ATO	phase separation	mild phase separation
Imwitor 900K	homogenous	homogenous
Softisan 154	mild phase separation	mild phase separation
Dynasan 118	homogenous	homogenous
Cocoa butter	homogenous	homogenous
Witepsol H15	homogenous	homogenous
Witepsol E85	phase separation	phase separation

© 2020 by the authors. Submitted for possible open access publication under the terms and conditions of the Creative Commons Attribution (CC BY) license (http://creativecommons.org/licenses/by/4.0/).
